# Assessing the unintended health impacts of road transport policies and interventions: translating research evidence for use in policy and practice

**DOI:** 10.1186/1471-2458-8-339

**Published:** 2008-09-30

**Authors:** Hilary Thomson, Ruth Jepson, Fintan Hurley, Margaret Douglas

**Affiliations:** 1 MRC Social & Public Health Sciences Unit, Glasgow, UK; 2 Department of Nursing and Midwifery, University of Stirling, Stirling, UK; 3 Institute of Occupational Medicine, Edinburgh, UK; 4 NHS Lothian, Edinburgh, UK

## Abstract

**Background:**

Transport and its links to health and health inequalities suggest that it is important to assess both the direct and unintended indirect health and related impacts of transport initiatives and policies. Health Impact Assessment (HIA) provides a framework to assess the possible health impacts of interventions such as transport. Policymakers and practitioners need access to well conducted research syntheses if research evidence is to be used to inform these assessments. The predictive validity of HIA depends heavily on the use and careful interpretation of supporting empirical evidence. Reviewing and digesting the vast volume and diversity of evidence in a field such as transport is likely to be beyond the scope of most HIAs. Collaborations between HIA practitioners and specialist reviewers to develop syntheses of best available evidence applied specifically to HIA could promote the use of evidence in practice.

**Methods:**

Best available research evidence was synthesised using the principles of systematic review. The synthesis was developed to reflect the needs of HIA practitioners and policymakers.

**Results:**

Aside from injury reduction measures, there is very little empirical data on the impact of road transport interventions. The possibility of impacts on a diverse range of outcomes and differential impacts across groups, make it difficult to assess overall benefit and harm. In addition, multiple mediating factors in the pathways between transport and hypothesised health impacts further complicate prospective assessment of impacts. Informed by the synthesis, a framework of questions was developed to help HIA practitioners identify the key questions which need to be considered in transport HIA.

**Conclusion:**

Principles of systematic review are valuable in producing syntheses of best available evidence for use in HIA practice. Assessment of the health impacts of transport interventions is characterised by much uncertainty, competing values, and differential or conflicting impacts for different population groups at a local or wider level. These are issues pertinent to the value of HIA generally. While uncertainty needs explicit acknowledgement in HIA, there is still scope for best available evidence to inform the development of healthy public policy.

## Background

Transport is often cited as an important determinant of health [1] and health inequalities, [2] and as such transport policies and interventions should be assessed for their potential to impact positively or negatively on health [2]. Physical injury and death are the most direct health impacts of motorised transport. However, other links between transport and health determinants need to be considered if the full potential for healthy transport policy is to be realised [1,3]. The possible impacts cover a range of important public health interests. These include physical activity and obesity, mental health, air quality and cardio-respiratory health, social exclusion and inequalities, and environmental impacts related to fuel emissions and climate change.

Health Impact Assessment (HIA) provides a helpful framework with which to assess the intended and unintended health impacts of policies or interventions. However, the validity of HIA depends substantially on the careful use and interpretation of supporting evidence. Shaping policies or interventions to maximise the potential health benefits and minimise adverse health impacts needs to be supported by empirical evidence, [4-6] but fresh comprehensive reviews of up-to-date evidence are beyond the scope of most individual projects and HIAs. Reviews of research, including systematic reviews, have previously summarised evidence of the health impacts of public policies such as transport and housing; but it cannot be assumed that their findings, often published in academic journals, will be transferred into practice. Well conducted syntheses of best available evidence informed by the needs of potential evidence users may facilitate knowledge transfer from research to practice [7]. Such syntheses need to draw on best available evidence from both intervention studies and epidemiological studies, [8] and to minimise author bias it may be valuable to apply the principles of systematic review, i.e. an explicit search strategy and assessment of the weight of evidence [9]. In addition, to promote the use of evidence in HIA practice, the relevance of the evidence to HIA needs to be made clear.

In 2003 we produced a synthesis of housing research in response to a request from a group of potential evidence users, the Scottish Health Impact Assessment Network (SHIAN-a multi-disciplinary group which consists of policy makers and practitioners from local and national government, and local health boards) [10,11]. This work drew on a systematic review of housing improvement and health as well as summarising the epidemiological links between housing and health. Following dissemination of the housing report, [11] the network identified transport as a priority area for a similar synthesis. This paper presents a summary of the research included in the synthesis of transport research and, informed by the synthesis, a list of key questions which need to be addressed when conducting an HIA of transport interventions. The synthesis is presented here as a demonstration of knowledge transfer to promote the use of evidence in HIA. We encountered a number of wider issues, e.g. lack of evidence, conflicting values, multiple outcomes, and differential impacts, throughout this work and we have used our experience to reflect on the implications for the development of evidence informed healthy public policy and HIAs of complex social interventions such as transport.

## Methods

### Communication with evidence users & scope of research synthesis

SHIAN members were consulted to identify topic areas and key questions to be covered in the synthesis [Appendix 1]. This informed the scope of the synthesis, which was then agreed in discussion with a sub-group of SHIAN. The review covered all major transport modes, road and non-road. [Table 1] For the purposes of this paper the expression 'transport intervention' denotes any transport policy, programme, or project. The health impacts of predicted climate change attributed to increased transport fuel use, transport policies for freight movement, or the health impacts of leisure or sport pursuits which use transport modes e.g. mountain biking, rally driving, were not included. The synthesis aimed to reflect SHIAN's key interest in the possible unintended health impacts of transport interventions rather than focus on the primary effectiveness of measures for reducing injuries. Outcomes included in the synthesis were identified by SHIAN [Table 1]. Specific health outcomes included were; injury and death, general health and illness, physical fitness and physical activity, and mental health (including stress). Factors considered by SHIAN to be possible determinants of health included air and noise pollution, personal safety, community severance (defined as reduced access to local amenities and disruption of social networks caused by a road running through the community) and social exclusion.

**Table 1:** Scope of and outcomes included in transport and health research synthesis

**Transport modes included**	All (N.B. Very little research evidence is available on the health impacts of non-road transport. This paper only reports on road transport)
**Topics not included**	Climate change attributed to increased motorised transport
	Transport policies for freight movement
	Health impacts of leisure or sport pursuits which use transport modes e.g. mountain biking, rally driving

**Health outcomes included in synthesis**	Injury & death
	General health & illness
	Mental health & stress
	Physical fitness & physical activity

**Non-health outcomes included in synthesis**	Air pollution
	Noise pollution
	Community severance
	Personal safety
	Social exclusion

### Search strategy & study inclusion/exclusion

Searches were carried out in 2006. We used a systematic review of systematic reviews (1960–2001) on transport and health [12] as a baseline resource and updated searches for systematic reviews published since 2001 (2001–2006). We searched ten bibliographic databases (Cochrane Library, DARE, SIGLE, PsycINFO, Medline, EmBase, SPORTDiscus, Cinahl, TRIS, and TRANSPORT) and the internet (Google) for systematic reviews of transport and health. Where no systematic reviews of an intervention were located, primary studies were searched for. Cross-sectional data on the associations between transport and health were identified from the above searches and an additional search on Web of Knowledge. All empirical studies identified were included (for a full list of studies see [13]) and the final synthesis reflects the relative strength of evidence of the identified studies [14,15]. Expert reviews were the main source of evidence on the health impacts of transport-related air pollution.

Since 2006 key journals have been hand-searched for relevant studies and reviews, in addition a final search for relevant systematic reviews was conducted in July 2008 in TRIS and the Cochrane Library (Issue 3).

### Synthesis and appraisal

A narrative and tabular summary of the research reviewed was prepared in light of the strength of evidence [see Additional files 1, 2, 3, 4]. An indication of the strength of evidence [Appendix 2] based on quality criteria for systematic reviews [14] and/or primary studies where appropriate was included in the summary tables (see Additional files) [15].

## Results

The following presents a summary of the full synthesis [13]. In this paper we are not able to report on every study included in the full synthesis, however, the key findings are presented in light of the quantity and quality of available data. Data on all included outcomes are presented where available. Very few studies of the health impacts of non-road transport were identified, but all identified studies of road and non-road transport were included in the final synthesis presented [13]. This paper reports on road transport and where available evidence on all transport modes using roads, for example trams, cycles, has been included.

### The Health Impacts of Road Transport Interventions

This first section summarises evidence on the health impacts of road transport interventions. The evidence draws on intervention studies, and the scope of interventions covered reflect the data identified by the searches.

### Interventions to reduce road transport injury

Injury reduction dominates transport and health research [12]. All but three of the systematic reviews we identified reported on injury reduction interventions.

#### Impact on injuries

A wide range of legislative, environmental, and safety equipment measures have been shown to lead to reductions in road injuries [see Additional file 2] [12,16,17]. Educational campaigns among the general population to promote the use of safety equipment, such as bicycle and motorcycle helmet, and children's car seats typically include education, incentives and/or distribution of free equipment. These campaigns have led to increased use of equipment such as cycle helmets and car seats, but little is known about subsequent impacts on injuries or other health outcomes [12,18,19]. Driver improvement and education courses may improve knowledge and safety behaviour, and may reduce crash involvement in some groups [20]. However, educational programmes to rehabilitate convicted drivers and high school driver education programmes are associated with increases in crash involvement and violations [12].

#### Other health impacts

One study of injury reduction measures had assessed a health related outcome which was unrelated to injury or accident outcomes. In this uncontrolled study a small improvement in physical health, but not mental health (SF-36), was reported and pedestrian activity was greater 6 months after the neighbourhood traffic calming measures were introduced [21].

### Interventions to promote physically active road transport: Promoting walking and cycling as an alternative to car use

Two systematic reviews related to this topic were found. They reviewed studies which had assessed the effectiveness of interventions to promote a modal shift from car use to walking and cycling, (a summary of reported impacts is provided in Additional file 3) [22,23].

#### Impact on physical activity (walking and cycling) & physical fitness

Programmes which target already motivated individuals may be effective at shifting up to 5% of trips from cars to walking and/or cycling. However, effects of similar programmes on the general, less motivated, population are unclear [22,23].

Other interventions which have been evaluated are: publicity and education aimed at the general population; financial incentives (road tolls, work subsidy for not driving to work); improved public transport; and car pools. From the research evidence available, there is very little to suggest that these interventions lead to a shift from car use to more active forms of transport.

It cannot be assumed that a shift from car use to more physically active forms of transport will automatically lead to an increase in overall levels of physical fitness or activity. For example, gym exercise may be replaced by cycling to work. However, one study assessed changes in fitness among those who changed from driving to walking or cycling to work; levels of fitness and walking speed improved [24,25].

#### Impact on general health & wellbeing

One study assessed the effects on general health for those who switched from driving to walking or cycling to work; there were significant improvements in general and mental health (SF-36) [26].

#### Other impacts: Injury, noise & air pollution

We found no available data on the injury or pollution impacts of interventions to promote a switch from car use to more physically active forms of transport. However, given the unclear effects of these interventions to achieve a significant modal shift, impacts on injury, noise and air pollution at a population level are likely to be minor.

### New road transport infrastructure: new or improved/upgraded roads

One systematic review was identified which had assessed the health impacts of new or improved roads (a summary of reported impacts is provided in Additional file 4) [27]. No research was identified which had assessed the health or health related impacts of other types of new road transport infrastructure, such as a tram network, or a new bus terminus.

#### Impact on injury

Provision of new or improved roads is likely to increase traffic volume. Nevertheless, nine of the 10 evaluation studies identified reported a fall in overall numbers of accidents and related injury [22]. Building by-passes to relieve traffic from urban areas may displace injury accidents from the old route to other secondary roads if smaller side roads are used as popular short-cuts, though the overall level of injury accident is still likely to fall.

#### Impact on respiratory health

One study assessed changes in respiratory symptoms after the opening of a bypass and an associated fall in pollutant levels in the by-passed street. While reports of rhinitis and rhino-conjunctivitis fell, there was little change in lower respiratory symptoms when compared to changes in a similar near-by street [28]. However, a small-scale intervention study such as this is unable to detect the main relationships between traffic-related air pollution and health.

#### Impacts on other possible determinants of health: noise, vibrations, fumes and dirt

New major urban roads are likely to result in increased levels of noise in the immediate vicinity. In some cases perceived traffic disturbance will improve as residents adapt to the changes, but this cannot be assumed. Conversely, where the new road diverts traffic from one road to another, those living in the area with reduced traffic are likely to experience fewer disturbances from noise, vibrations, fumes and dirt.

#### Impacts on other possible determinants of health: community severance

There is very little research evidence on the impacts of new roads on community severance. One US study reported a reduction in the number of people crossing a new road running through a neighbourhood and that this effect was still observable 30 years later [29]. Where a new road leads to reduced traffic on by-passed roads, the severance effect will be reduced [27].

#### Impact displacement and volume

Although a new road may reduce traffic volume on some roads, e.g. through a town centre, it is unlikely that overall traffic volume will be reduced indeed improved road provision may lead to increased traffic overall (i.e. induced traffic). In the case of bypasses, traffic and its associated impacts, i.e. air pollution, will likely be displaced and increase on other roads, in particular the bypass area itself. Some studies have looked at the overall impacts of new roads on injuries reporting an overall decrease but there is little detailed reporting of the distribution of impacts. Even if the overall impact is clear e.g. reduced injuries, there may be small pockets which experience increased traffic due motorists detouring through quieter, often residential, streets to avoid congestion or traffic control measures, also known as 'rat-running'.

### The health impacts of reducing road transport noise pollution

Interventions to reduce road noise include eliminating noisy vehicles, reducing traffic speed, and developing quieter road surfaces e.g. porous asphalt [30]. There is little research evidence about the health impacts of effective measures to reduce traffic noise, but reduced traffic noise may reduce sleep disturbance.

### The health impact of interventions to reduce road transport related air pollution

Interventions to reduce air pollution from motor vehicles in the UK include unleaded petrol, low sulphur fuels, and various European directives to control emissions of particles and oxides of nitrogen. These measures have led to clear reductions in air pollution; impacts on health however have been inferred rather than studied directly [31]. A review of interventions that reduced air pollution identified two studies which had assessed the health effects of policies specifically designed to reduce transport-related air pollution [32].

#### Impact on air pollution

High-sulphur fuels were banned in Hong Kong in 1990, leading to an immediate, marked and sustained decrease in ambient SO_2_, with changes also to the surface characteristics of fine particles [33]. Short term traffic restriction measures were introduced over a 17-day period during the Atlanta (US) Summer Olympic Games of 1996, with significant reductions in levels of carbon monoxide, particulate matter (PM_10_) and ozone within the affected area [34].

#### Impact on cardio-respiratory health & mortality

Prior to the banning of high sulphur fuels in Hong Kong, monthly deaths were rising by 3.5% per year due to demographic changes. The five years following the intervention showed a clear, immediate and sustained reduction in the rate of increase in mortality. The change was greatest for pollution-related causes, i.e. cardio-respiratory, and occurred in the high SO_2 _reduction areas; the low SO_2 _reduction areas showed a higher increase in mortality after the intervention than before [33]. Following the traffic restrictions in Atlanta there was a small reduction in the number of asthma events requiring hospital attention among children, when compared with 4 week period before and after the games. There was no change in the number of children requiring acute care due to other causes [34].

### Interventions to reduce road traffic: Congestion charging

Two studies of traffic restriction measures were identified: one study of the London Congestion Charging (LCC) scheme; [35] the second study was of short-term traffic restrictions scheme during the Atlanta Olympic games which partly aimed to reduce air pollution and is reviewed above (section I (e)) [34].

#### Impacts on injuries

There is no evidence of an increase in serious road injuries and it is estimated that between 40–70 crashes per year have been prevented in the zone area [35].

#### Impacts on air & noise pollution

While there is some suggestion that air quality has improved in the LCC zone, the monitoring programme of the LCC scheme reports that it has not been possible to detect a 'congestion charging effect' in measures of air quality [35]. There are no reports of changes in traffic noise.

#### Other impacts: local congestion & economy

Two years after the introduction of the LCC scheme traffic entering the zone is reduced by 18%, and traffic speeds increased due to reduced congestion. Those living within the charging zone report little change in their car use [35].

There does not appear to have been any impact on local economic outcomes such as business performance, employment, property prices and retail sales [35].

### The health impacts of improving negative psycho-social impacts of public road transport

We were unable to identify much research in this area. One systematic review of the crime prevention effects of closed circuit television (CCTV) included four evaluations of CCTV on public transport. Results were mixed and the pooled effect, a 6% reduction in crime, was not significant [36].

### Associational Evidence Supporting Links Between Road Transport and Health and Possible Determinants of Health

In addition to synthesizing data on the health impact of interventions, we searched for research evidence to support the hypothesized links between different modes of transport to health and other health related factors specified by SHIAN. While these data report associations between transport and health, the direction of the relationship is rarely clear, and evidence of an association does not imply a causal relationship. A summary of the data, with an indication of the strength of the association, is presented in Additional file 1.

#### General health

Car ownership and access has been associated with better health and fewer long term health problems [37]. This association may be explained by the relationship between car ownership and socio-economic status, but two studies report that the association persists even when income, social class and self-esteem are controlled for [38,39].

#### Mental health & stress

Access to a car has been associated with improved mental health in two studies in Scotland. This association was shown to be independent of social class, income, and self-esteem [38,39].

Physically active forms of transport may lead to increased overall levels of physical activity. For individuals who achieve significant increases in physical activity there may be small mental health benefits [40,41].

Commuting to work, by road and by train, is associated with increased stress and short term elevations in blood pressure. Shorter, familiar, and convenient (i.e. direct route) journeys may be less likely to cause increased stress. The long term health impact of frequent commuting is not known [42,43].

#### Physical injury & death

Despite massive increases in motorized road traffic, in most industrialized countries (UK data 1980–2004 +80% [44]) the rates of road casualties and absolute numbers of fatalities have been falling for the past 50 years [45,46]. Motorised road traffic exposes more vulnerable road users, namely cyclists and pedestrians, to a hugely increased risk of injury. However, this risk may vary between countries; the risk to cyclists appears to be inversely related to the proportion of cyclists on the road. Evidence from Holland, Denmark, and Germany suggest that a critical mass of cyclists on the roads leads to a reduced risk of cyclist injury despite an increase in cyclist miles travelled [47,48]. In addition, countries where cyclists are accepted as co-road users a wide range of measures, education and engineering measures, are implemented to promote cyclist safety [49].

Trams were an area of interest to SHIAN, but we were unable to locate much research around the health impacts of tram systems. Two identified studies suggest that cyclists are most at risk from trams, [50,51] in one study the most common scenario being where cycle wheels become trapped in the tram tracks [50].

#### Physical activity & fitness

Clearly certain forms of transport involve more physical activity than others, but this cannot be assumed to affect overall levels of physical activity or indeed levels of physical and cardiac fitness. Very little data are available at an individual level to link car use and overall physical activity levels. While there are some data to suggest that children in families who are 'highly car dependent' may be less physically active, [52] another study reports that car ownership is associated with increased levels of physically active leisure independent of socio-economic status [53]. A further study reported a link between time spent in cars and obesity [54].

Physical characteristics of the local environment have been associated with levels of physical activity and physically active transport [55,56]. For example, physically active transport (i.e. walking or cycling) has been directly related to increased residential density, street connectivity, mixed land use and amenities within a walkable distance [57]. Where using public transport involves walking to and from a transit point this may help otherwise inactive groups to increase their levels of walking [58]. An economic analysis has suggested that the potential increase in energy expenditure could lead to significant savings on obesity related medical costs in the long term [59].

#### Air pollution

Transport fuel emissions contribute directly to air pollution which has a direct impact on cardio-respiratory health [60] and methodologies for including air pollution effects in HIA are well developed [31]. The most significant public health effect is an increased risk of mortality from long-term exposure to fine ambient particles (PM_2.5_) [61]. There is specific evidence of increased risk of mortality [62] and morbidity [63] in people living near major roads; the Dutch mortality study [62], for example, found that deaths from cardio-respiratory causes were almost twice as likely (relative risk 1.95; CI 1.09–3.52) in people who had lived within 50 metres of a major road for 10 years or more. Factors other than transport-related air pollution may have contributed to the increases in risk. The health effects of transport-related air pollution were reviewed recently by the WHO [64].

Reports from the UK Air Quality Expert Group (AQEG) imply that traffic is responsible for about half of the overall PM_2.5 _in the UK [65,66]. On that basis, transport-related air pollution (PM_2.5_) is estimated to reduce life expectancy by a few months, an effect similar to, or a little greater than, the estimated effect of passive smoking [67].

#### Noise pollution

Noise from road intersections above 50–60 dB(A) is insufficient to lead to hearing loss but has been reported to cause annoyance and sleep disturbance; impacts on other long term health outcomes including blood pressure are less clear [68].

#### Community severance

No empirical data reporting a link between community severance and health were identified and the possible health impacts of remain unknown.

### Other Considerations

Predictions of health impacts need to be considered in light of the broader aim of transport and different transport needs which may vary by country, local area, population sub-group, and individual.

#### Transport & access as a health determinant

The primary function of transport is the movement of people and goods between places, enabling access to employment, economic, and social opportunities as well as to essential services. Transport needs will depend on many local contextual factors e.g. existing public transport, rurality, as well as individual factors, e.g. mobility. But transport which is affordable and accessible may be viewed as an important determinant of health by facilitating access to key socio-economic opportunities.

#### Transport & social exclusion/inequalities

Inadequate transport provision may add to social exclusion among already vulnerable groups, i.e. those who are unemployed, elderly, sick, on low incomes, and women, presenting a barrier to jobs, health services, education, shops and other services [69,70].

Lack of access to a car may contribute to transport related social exclusion [70-72]. In the UK, car ownership is strongly associated with income, yet the association between car ownership and improved health is independent of income and social class. This may be explained by the improved access that a car provides [38,39].

Disadvantaged groups are least likely to own a car, compounding disadvantage in a car-dominated society. Yet, ironically, the same groups experience a disproportionate amount of the harmful effects of cars. Children from the poorest households are between four and five times more likely to be killed in a road traffic accident than their counterparts from the most affluent households [73].

#### Determinants of transport mode

When considering the potential for a shift in transport behaviour it is essential to consider the reasons, other than health, for choosing different modes of transport. In particular, the considerable positive benefits (convenience, time, comfort, personal safety, carrying loads, and costs (for existing car owners)) reported to be linked to car use compared to all other transport modes [74].

#### Health impacts of road transport related climate change

It is estimated that transport-related fuel use accounts for around 22% of CO_2 _fuel emissions [75]. Although individual fuel use may have a negligible impact, an accumulation of increased fuel emissions may have significant environmental, economic and health impacts at a global level.

The balance of health impacts related to climate change is likely to be adverse, particularly in the developing world. The WHO estimates that climate change has already caused 150,000 deaths [76].

#### Applying evidence to policy & practice

Informed by the synthesis, we produced a list of questions [Table 2] which may be used as a guide to shape assessments of the potential health impacts of a planned transport intervention or policy. In addition to questions directing assessors to consider the empirical support for predicted impacts, Table 2 includes questions central to defining the scope of the HIA and the actual intervention and population being included.

**Table 2:** Questions to help shape HIAs of road transport interventions

*Define nature and extent of intervention or policy being assessed*
• What are the specific transport-related changes proposed?
• What is/are the overall aim(s) and objectives of the transport changes proposed?
• How will the changes be implemented?
• What phases of implementation are there, e.g. consultation, implementation/construction, maintenance?

***Research evidence about health impacts of the intervention***

• What is the research evidence that this intervention is effective in achieving its stated aims e.g. reducing speed?
• What is the research evidence that this intervention will have the intended health impacts (positive or negative)? Include any stated health objectives of the intervention.
• What is the research evidence that this intervention has unintended health related impacts (positive or negative)?

***Define features of the local area***

• What is/are the geographical area(s) covered by the intervention?
• What are the key features of the area:
• Is it urban or rural?
• What transport infrastructure currently exists?
• What facilities and amenities are there that people need to access?

***Define populations***

• What populations will be affected by the changes?
• Note any vulnerable population groups.
• For each impact identified who will be affected positively.
• For each impact identified who will be affected negatively.
• Will the impacts be distributed equally in difference socio-economic groups? *If not this may have implications for health and social inequalities*.

***Economic implications***

• What are the predicted effects of the proposal on the local economy?
• How will travel costs be affected for individuals?

***Changes in travel and traffic patterns***

• How will traffic levels or speed change? If appropriate, consider different parts of the affected area separately.
• Where relevant, will improved provision lead to increases in overall Vehicle Miles Travelled (VMT) i.e. induced traffic?
• Will there be any part of the affected area where traffic levels, speed, or infrastructure, will change to the extent that severance effects may occur?
• How will these changes affect access to essential services and amenities for those living in or travelling through the affected area?
• What will be the effect on individuals' travel patterns? Consider levels of driving, walking, cycling, and public transport use. Consider travel patterns of those both living in and travelling through the affected area(s).
• How will the expected changes affect safety for vehicle drivers or other transport users?
• How will the expected changes affect safety for other vulnerable road users, e.g. pedestrians?
• How will the expected changes affect air quality in the affected area?
• How will the expected changes affect noise levels in the affected area?
• Will there be a shift to more or less physically active forms of transport? (Walking, cycling or public transport use)
• Will this shift affect individuals' levels of physical activity overall?
• Will this change in physical activity be sufficient to affect health?
• Will changed levels of physical activity be seen in the general population of the affected area or in a minority of motivated individuals?
• How will safety, and perceptions of safety, among vulnerable road users and public transport users be affected?

***Traffic and impact displacement***

• Will there be displacement of traffic and related impacts to or from surrounding areas? For example, traffic calming may lead to less traffic in one area but displace traffic to a peripheral area. *If displacement is expected a Health Impact Assessment should consider impacts on both areas*.

## Discussion

This evidence synthesis aims to provide a digest of the best available evidence within the transport and health field for use by public health policymakers and practitioners. Where available we endeavoured to meet the evidence needs of SHIAN [Table 2], but for many of their questions there was no evidence available. While drawing heavily on systematic reviews, other types of research, including single intervention and cross-sectional studies, have also been reviewed [77]. The principles of systematic review were applied to the synthesis in order to minimise bias in the data selected and so that the digest of research reflected the relative strength of evidence with respect to study quality. The list of questions [Table 2] aims to assist discussion and assessment of the health impacts of transport interventions, and the figure in Additional file 5 demonstrates how the evidence synthesis might be used to populate theoretical pathways for predicted impacts of specific interventions. Far from presenting a clear map of health impacts, many of the impacts included in this review are characterised by uncertainty. This has important implications for the potential value of transport HIA [Table 3] and highlights the need for accurate assessment and representation of uncertainty within transport HIAs.

**Table 3:** Some key issues affecting the predictive value of transport HIA

• Multiple outcomes present conflicting overall benefit and harm at different levels
• Lack of empirical support for plausibility of links to actual health impacts
• Numerous steps and mediating factors influence links between transport and health
• Defining a transport intervention and affected area and population not always straightforward

### Wide range of possible impacts

The links between transport and health cover a vast literature on diverse transport modes, and a variety of issues important to public health. However, while there is a considerable literature on the direct impacts of transport on injury there is far less to forecast other unintended health impacts which are central to HIA.

This wide range of possible impacts means that policies may be beneficial in some respects and harmful in others. There may also be differential and conflicting impacts depending on the level (individual v population), location, and timescale of measurement. This adds further to the potential for conflict between impacts and also increases uncertainty around overall benefits and harms. Supplemental Figure 1 (see Additional file 5) illustrates some mediating factors and conflicting benefits and harms which might follow a modal shift to active commuting. This mix of benefits and harms requires difficult decisions about which outcomes and population groups to prioritise [78]. This may be partly resolved by using a common metric to represent diverse health outcomes, for example Quality-Adjusted Life Years (QALYs) [79] or monetary valuation, [31] however such an approach may conceal differential impacts.

### Empirical support for plausibility of predicted impacts & their pathways

The value of HIA depends largely on the accuracy with which it can correctly predict future impacts. While it is increasingly accepted that predictive validity, based on empirical data from intervention studies, is unlikely to be available to HIA, plausibility and formal validity supported by best available scientific evidence is desirable [77]. This involves setting out plausible pathways for predicted impacts (e.g. Figure S1-see Additional file 5) and gathering empirical support for each step in the pathway. For the types of interventions subject to an HIA the best available evidence is likely to come from both intervention and cross-sectional studies. Indeed, data from cross-sectional studies may in some cases be superior in terms of both quality and quantity, such as when modelling the health impacts of transport-related air pollution.

While each of the hypothesised impacts and pathways in this synthesis was regarded as plausible it is disappointing that we found so little empirical support. Much of this uncertainty owes to lack of evidence rather than evidence of no effect, either from intervention or cross-sectional studies.

The hypothesised pathways linking a transport intervention to a possible health impact will often involve more than one step; between each step there are numerous mediating factors. For example, at an individual level there are many influences on transport choices, such as cost, time, weather, safety, passengers [74]. At a wider level, large-scale transport interventions cannot be separated from the local and political context within which they occur [80]. Even with stronger empirical support for specific impacts, these numerous mediating factors introduce an inevitable and substantial amount of uncertainty to the development of health-related transport policy; uncertainty which should be clearly acknowledged in transport HIAs.

### Defining transport interventions, affected area(s), and affected population(s)

Further challenges lie in defining the intervention(s), identifying the geographical areas and population(s) affected (a helpful description of attempts to define a motorway extension and a congestion charging scheme is presented by Ogilvie et al 2006) [80]. Structural transport interventions may lead to traffic displacement. For example, a bypass will reduce traffic through a town but may increase traffic around the bypass and may result in 'rat-running' on residential roads by drivers trying to find short-cuts. There may also be differential impacts across areas and population groups. For example, noise effects are necessarily close to source, whereas transport air-pollution may have long-range effects. Differential impacts across socio-economic groups raise further issues of equity. For example, in a context of growing car dependence, financial incentives to reduce car use will be disproportionately harsh on low-income groups, and may increase social exclusion and subsequent health inequalities. Conversely, subsidies to promote a modal shift from private car to public transport may be of great benefit to those on low incomes. Highlighting differential impacts, including unintended consequences, is a central element of HIA [81].

## Conclusion

When compared to a similar synthesis of the health impacts of housing improvement, [10] the uncertainty and complexity in attributing health impacts to transport interventions appears to be much greater. Injuries and deaths caused by motor-vehicles are indisputable and already closely monitored with many effective interventions in place to minimise this harm. The strength of evidence about other indirect health related impacts varies according to the pathways concerned, from strong quantifiable evidence of air pollution effects, to much weaker evidence on the health effects of transport noise and community severance. This leads to considerable uncertainty in assessing the overall benefits and harms of transport interventions.

However, few decisions, in policy or elsewhere, are supported by thorough knowledge or conclusive outcome evaluations. And lack of conclusive evidence does not preclude the possibility for small increases in risks across a large population to have significant public health impacts. It remains that transport interventions have important potential impacts on health and health inequalities. While HIA practitioners need to make the inevitable uncertainties explicit in their assessments, HIA has a valuable role to play in raising awareness of the potential impacts, and to inform the development of healthy public policy.

## List of abbreviations

SHIAN: Scottish Health Impact Assessment Network; US: United States (of America); UK: United Kingdom; SF-36: Short Form 36 item questionnaire; CCTV: Closed Circuit Television; LCC: London Congestion Charging

## Competing interests

The authors declare that they have no competing interests.

## Authors' contributions

RJ & HT led on the evidence synthesis: FH contributed a synthesis of evidence on transport related air pollutants; MD prepared the section on transport related climate change; RJ & HT prepared all other sections of the synthesis. HT developed the questions in Table 2. HT prepared the first draft of the paper and all authors contributed to subsequent drafts.

## Appendix 1: Questions to be addressed by research synthesis (as proposed by SHIAN)

• What is the evidence that transport policies and initiatives can affect physical activity levels overall? (taking account of, eg, substitution effects)?

• What is the evidence that transport policies and initiatives can affect road safety for car drivers, passengers and pedestrians?

• What is the evidence of health effects from air and noise pollution from different modes of transport? What population subgroups are affected?

• What is the evidence of links between stress and mode of travel?

• What is the evidence of impacts of transport policies and initiatives on community severance, and resulting impacts on health? This would include, eg, new roads, crossings, how busy roads are.

• What is the evidence of links between social inclusion and transport policies and initiatives?

• What is the evidence of health impacts of initiatives intended to effect modal shift?

• What is the evidence of the direct and indirect health impacts of measures to promote availability and use of public transport?

• What are the most effective interventions for:

• reducing drink driving?

• reducing speed?

• increasing seat belt use?

• increasing helmet use?

## Appendix 2: Classification used for Strength of Evidence (SoE)(adapted from Weightman et al 2005 [15])

1++ High quality meta-analysis, systematic review(s) of RCTs (including cluster RCTs) or RCTs with a very low risk.

1+ Well conducted meta-analysis, systematic review of RCTs, or RCTs with a low risk of bias.

1- Meta-analysis, systematic reviews of RCTs, or RCTs with a high risk of bias.

2++ High quality systematic reviews of, or individual high quality non-randomised intervention studies (controlled non-randomised trial, controlled before-and-after, interrupted time series) comparative cohort and correlation studies with a low risk of confounding, bias or chance.

2+ Well conducted, non-randomised intervention studies (controlled non-randomised trial, controlled before-and-after, interrupted time series), comparative cohort and correlation studies with a low risk of confounding, bias or chance.

2- Systematic review (Oxman & Guyatt score < 5: moderate to poor quality)[14] of non-randomised intervention studies with high risk of confounding, bias or chance. Non-randomised intervention studies (controlled non-randomised trial, controlled before-and-after, interrupted time series), comparative cohort and correlation studies with a high risk of confounding, bias or chance.

3 Non-analytical studies (e.g. case reports, case series), single cross-sectional study or single small non-randomised intervention study (controlled non-randomised trial, controlled before-and-after, interrupted time series), comparative cohort and correlation studies with a high risk of confounding, bias or chance.

4 Expert opinion, formal consensus

## Pre-publication history

The pre-publication history for this paper can be accessed here:



## Supplementary Material

Additional file 1 **Table S1.** Summary of hypothesised links between road transport and health with Strength of Evidence (SoE). Click here for file

Additional file 2 **Table S2.** Overview of health impacts of interventions which aim to reduce transport related injury with Strength of Evidence (SoE). Click here for file

Additional file 3 **Table S3.** Summary of health and environmental impacts of initiatives promoting physically active forms of transport with indication of Strength of Evidence (SoE). Click here for file

Additional file 4 **Table S4.** Summary of the health and related impacts of new roads with indication of strength of research evidence (SoE). Click here for file

Additional file 5 **Figure S1.** Some possible pathways to health and related impacts following modal shift from driving to cycling to work. Click here for file
