# Supplementary material for: Assessing the unintended health impacts of road transport policies and interventions: translating research evidence for use in policy and practice
Source: BMC Public Health. 2008 Sep 30;8:339. doi: 10.1186/1471-2458-8-339 (PMC2567981; doi:10.1186/1471-2458-8-339)
Supplement: Additional file 1 — Table S1. Summary of hypothesised links between road transport and health with Strength of Evidence (SoE). [file 1471-2458-8-339-S1.doc]

***Table 1: Summary of hypothesised links between road transport and health with Strength of Evidence (SoE)*** *^[1]^ see Appendix 2*

| **Transport factor** | **General health** | **SoE** | | **Mental health & stress** | **SoE** | **Physical injury/death** | **SoE** | **Physical activity** | **SoE** |
| --- | --- | --- | --- | --- | --- | --- | --- | --- | --- |
| **Mode of transport** | | | | | | | | | |
| **Car** | Very little research investigating links between use of different transport modes and general health | |  | Very little research investigating links between use of different transport modes and mental health |  | 3.7 KSI* per million kilometres travelled ^[2]^ |  | Sedentary form of transport |  |
| **Pedal bike/motorcycle** |  |  |  |  |  | 53.3/119 KSI* per million kilometres travelled ^[2]^ |  | Physically active form of transport |  |
| **Foot** |  |  |  |  |  | 1.4 KSI* per million kilometres travelled  ^[2]^ |  | Physically active form of transport |  |
| **Public transport- Bus/Coach** |  |  |  |  |  | 7.2 KSI* per million kilometres travelled ^[2]^ |  | May require short walk to pick-up point ^[3]^ (mean time from home to bus-stop= 6 minutes in Scotland) ^[4]^ |  |
| **Car ownership/ Access** | Improved health independent of social class, income, self-esteem ^[5, 6]^ | 2- | | Improved mental health: independent of income and self-esteem ^[5, 6]^ | 2- |  |  | High levels of car use may be linked to lower levels of physical activity ^[7]^  Those with access to a car are more likely to participate in physically active leisure, independent of socio-economic status ^[8]^ | 2-  2- |
| **Road transport factors/impacts and hypothesised links to health or related social outcomes** | | | | | | | | | |
|  | **Links to health and related social outcomes supported by research evidence** | | | | | | | | **SoE** |
| **Physical activity** | Moderate physical activity, such as brisk walking, accumulating to 30 minutes on 5 days per week is recommended for adults to benefit health. Regular moderate activity may lead to reduced risk of chronic disease and death from any cause and may help control weight and prevent obesity. ^[9]^ | | | | | | | | 2++ |
|  | Physical activity may have a protective effect on mental health. ^[10]^ | | | | | | | | 2++ |
| **Community severance** | May disrupt local social networks and access to local services but potential for impact will vary substantially by area geography. Health impacts are unknown. ^[11]^ | | | | | | | | 3 |
| **Air pollution** | Traffic contributes to outdoor air pollution. Both short-term and longer-term exposure to ambient particulate matter (PM) increases the risks of death and disease from cardio-respiratory causes. Some effects are more-or-less immediate and affect vulnerable groups in particular whereas the effects of long-term exposure are more widespread. ^[12, 13]^ In Britain, long-term exposure to transport-related air pollution measured as PM_2.5_ is estimated to reduce life expectancy by a few months, an effect similar to the estimated effect of passive smoking. ^[14]^ | | | | | | | | 3 |
| **Noise pollution** | Not sufficient to lead to hearing loss, but is likely to cause sleep disturbance for those living in the immediate vicinity of a busy street or motorway. Other health effects are unknown. ^[15]^ | | | | | | | | 3 |
| **Personal safety** | May affect decisions to walk, cycle or use public transport but health effects are not known ^[16] [17]^ | | | | | | | | 3 |
| **Stress** | Traffic congestion may cause short term elevations in stress markers but possible long term effects are not known ^[18] [19]^ | | | | | | | | 2- |

*** KSI: Killed/seriously injured. Vehicle kilometres travelled (UK data 2005) Figures will vary by country factors and varying proportions of different transport modes e.g. cyclists ^[2]^**

**References**
