# Supplementary material for: Assessing the unintended health impacts of road transport policies and interventions: translating research evidence for use in policy and practice
Source: BMC Public Health. 2008 Sep 30;8:339. doi: 10.1186/1471-2458-8-339 (PMC2567981; doi:10.1186/1471-2458-8-339)
Supplement: Additional file 2 — Table S2. Overview of health impacts of interventions which aim to reduce transport related injury with Strength of Evidence (SoE). [file 1471-2458-8-339-S2.doc]

**Table 2: Overview of health impacts of interventions which aim to reduce transport related injury with Strength of Evidence (SoE)^[1]^ see Appendix 2**

| **Intervention** | **Impact on accidents in affected area** | **Effect on fatal and serious injury from accidents** | **Effect on less serious injury from accidents** | **Other health related effects** | **Other unintended**  **effects/comments** |
| --- | --- | --- | --- | --- | --- |
| **Environmental and engineering:** separating pedestrians from vehicles and increasing pedestrian visibility | | | | | |
| Exclusive pedestrian signalling (traffic light pedestrian crossings) ^[2]^ | Decreased (2-) |  |  |  | Effectiveness will depend on use which will depend on perceived safety and convenience for pedestrian users |
| Pedestrian overpasses and underpasses  ^[2]^ | Decreased (2-) |  |  |  |
| Median/refuge islands in multi-lane roads  ^[2]^ | Decreased (2-) |  |  |  |
| Pavements ^[2]^ | Decreased (2-) |  |  |  |
| Advance stopping lines (indicating vehicles to stop a few feet from crossing) ^[2]^ | Decreased (2-) |  |  |  |  |
| Street lighting^[3]^ | Decreased (2-) | Decreased (2-) |  |  |  |
| Diagonal on-street car parking ^[2]^ |  |  |  |  | Reduces number of pedestrians entering road in front of a parked vehicle (2-) |
| **Environmental and engineering:** managing vehicle speeds | | | | | |
| Speed limit zones, e.g. twenty’s plenty ^[4]^ |  |  | Decreased (2-) |  | Speed limit zones in quiet peripheral roads also lead to reduced amount of material damage |
| Changes to speed limits- slower in built up areas and faster on peripheral roads ^[4]^ | May increase accidents on peripheral roads where speed limit is increased (2-) |  |  |  |  |
| Roundabouts ^[2]^ | Decreased (2-) |  |  |  | Largest decreases in accidents observed when the intersection was previously controlled by a Give Way sign rather than by traffic lights |
| Road humps ^[4]^ | Unclear (2-) |  |  |  | May displace accidents to alternative local roads |
| Raised crossroads ^[4]^ | Small increase (2-) |  |  |  |  |
| Rumble strips ^[4]^ |  |  | Decreased (2-) |  | Reduced levels of material damage from traffic accidents (2-) |
| Area-wide traffic calming ^[4]^ | Decreased (2+) |  |  | Possible increase in walking in local area (2-) | Impact on noise levels will vary depending on type of traffic |

| **Environmental and engineering:** separating vehicles from other vehicles, pedestrians and local area (i.e. motorways running through an area) | | | | | |
| --- | --- | --- | --- | --- | --- |
| Guard rails ^[4]^ | Decreased (2-) | Decreased (2-) | Decreased (2-) |  |  |
| Crash cushions ^[4]^ | Decreased (2-) | Possible decrease (2-) | Possible decrease (2-) |  |  |
| Central reservation crash barriers ^[4]^ | Increased (2-) | Decreased (2-) | No change (2-) |  |  |
| **Legal strategies** |  |  |  |  |  |
| Blood alcohol concentration (0.08g/dl or 0.08%) ^[5]^  ^[4]^ | Decreased * (2+) |  | Decreased * (2+) |  | UK legal limit is 0.08% |
| Lower blood alcohol concentration for teenage drivers (0.02g/dl or 0.02%) ^[6]^ ^[5]^ | Decreased * (2+) | Decreased * &** (2+) |  |  |  |
| Minimum legal drinking age ^[5]^ |  | Decreased * (2+) | |  | Reduces drink driving among younger drivers |
| Random breath testing ^[5] [4]^ | Decreased * (2+) | Decreased * (2+) | |  |  |
| Red light cameras ^[7]^ | Unclear (2++) | Decreased (2++) | Decreased (2++) |  | Reduces red light running |
| Speed cameras ^[8]^ | Decreased (2++) | Decreased (2++) | Decreased (2++) |  | Reduces speed at affected area (2++) |
| Motorcycle helmet legislation ^[9]^ ^[10]^ |  | Decreased (2-) | Unclear effect on facial & neck injuries |  |  |
| Bicycle helmet legislation ^[11]^ | Decreased (2++) |  |  | Increased helmet use (2++) | Unknown impact on cycle use (2++) |
| Graduated licensing laws ^[12]^ | Decreased (2+) | Decreased (2+) | Decreased (2+) |  | *Not used in the UK* |
| Seatbelt legislation (primary) ^[13]^ |  | Decreased (2-) |  | ncreases seat belt use | I |
| Licence ban/suspension for problem drivers ^[14]^ | Small decrease (2+) |  |  |  | May also reduce rates of violation among problem drivers |
| **Safety equipment for individuals** | | | | | |
| Motorcycle helmets ^[15]^ ^[10]^ |  | Decreased head injury. Unclear effect on neck and facial injury. (2+) | |  | Impact dependent on speed and driving habits of helmet wearers |
| Bicycle helmets  (pedal cycle) ^[9]^ ^[16]^ |  | Decreased (2+) | Decreased (2+) |  | May not prevent lower facial injuries. |
| Visibility aids for pedestrians ^[17]^ |  |  |  |  | Improves drivers ability to identify and respond to vulnerable road users |
| Daytime running lights ^[4]^ | Decreased (2-) |  |  |  | *Not commonly used in the UK* |
| Studded tyres ^[4]^ | Unclear (2-) |  |  |  |
| Seatbelts ^[18]^ ^[19]^ ^[20]^ |  | Decreased (2++) |  |  | Potential for benefit depends on use of seatbelt |
| **Educational interventions** | | | | | |
| Post-license driver educational courses ^[21]^ | Mixed effects (2++) |  |  |  |  |
| Distribution of educational material to problem drivers ^[14]^ | No change (2++) | No change (2++) | No change |  |  |
| School based driver education ^[4]^ | Possible small increase (1+) | No change (1+) | No change (1+) |  | Leads to earlier licensing among teenagers |
| Road safety campaigns ^[4]^ |  |  |  | Improves road safety behaviour among pedestrians (2++) |  |
| Child safety campaigns (road behaviour) ^[4]^ ^[22]^ |  |  |  | Fewer children walk onto road from behind a parked car (2++) | Improves children road safety knowledge and behaviour |
| Promoting use of cycle helmets ^[4]^ ^[23]^ |  |  |  | Decrease in hospital admissions for cycle injuries (2++)  Increased helmet use |  |
| Counselling on use of child safety seats among general population of parents ^[24]^ |  |  |  | Increased use of equipment especially provision of free/reduced cost equipment (2++) |  |
| Counselling on use of car seat restraints among general population (children & adults) ^[24]^ |  |  |  | Unclear effect for adults and children (2++) |  |
| Counselling on drink driving among general population ^[24]^ |  |  |  |  | Insufficient evidence |
| Education +/- incentives/distribution of free child booster seats ^[24, 25]^ |  |  |  | Increased use of equipment especially provision of free/reduced cost equipment (2++) |  |
| Retraining older drivers ^[26]^ |  |  |  | Improved safe driving behaviour (2++) |  |
| Promoting seat belt use ^[27-29]^ |  | Unclear (2++) | Unclear (2++) | Increased use of seat belts and in-car restraints for children | Community based campaigns with financial incentives may be most effective- especially where seat belt use is already low |
| Reducing drink driving (mass media campaigns) ^[30]^ | Decreased* (2++) |  |  |  |  |
| **Remediation of drinking and driving offenders** | | | | | |
| Alcohol ignition interlock ^[31]^ |  |  |  |  | Reduces re-offending while lock fitted to car. (2++)  *Not used in the UK.* |
| Probation & rehabilitation ^[4]^ | Decreased (1+) | Decreased (1+) | |  | Probation and rehabilitation together may increase risk of injury (1+) |
| Treatment of convicted drivers ^[4]^ | Decreased *  Increased ** (1+) |  |  |  |  |
| Licence ban/sanction ^[4]^ | Increased (light  sanctions) (2-)  Decreased (severe sanctions) (2-) |  |  |  |  |

* alcohol related crashes

** non-alcohol related crashes

NB: Blank cells indicate that there is no available research evidence reporting on this specific impact.

Where the cells for serious and less serious injury are merged this indicates that available data has not distinguished between serious and non-serious injury

**References**
