# Supplementary material for: Assessing the unintended health impacts of road transport policies and interventions: translating research evidence for use in policy and practice
Source: BMC Public Health. 2008 Sep 30;8:339. doi: 10.1186/1471-2458-8-339 (PMC2567981; doi:10.1186/1471-2458-8-339)
Supplement: Additional file 3 — Table S3. Summary of health and environmental impacts of initiatives promoting physically active forms of transport with indication of Strength of Evidence (SoE). [file 1471-2458-8-339-S3.doc]

**Table 3: Summary of health and environmental impacts of initiatives promoting physically active forms of transport with indication of Strength of Evidence (SoE) ^[15]^ see Appendix 2**

|  | **Example of intervention** | **Walking & Cycling** | **SoE** | **Physical fitness & weight** | **SoE** | **General**  **wellbeing** | **SoE** | **Road traffic crashes**  **& injury** | **SoE** |
| --- | --- | --- | --- | --- | --- | --- | --- | --- | --- |
| **Engineering measures** | Road based measures to promote safe walking and cycling, e.g. cycle lanes, speed restrictions | Unclear effect | 2+ |  |  |  |  |  |  |
| **Targeted behaviour change** | Individualised marketing of alternative modes of transport to households showing an interest in using them | May shift up to 5% of car trips among motivated sub-groups  Increase in walking as form of transport up to one year later | 2+ | Small improvement in fitness  No change in average weight | 2+  2- | Possibility of small improvements | 2+ | No changes reported | 2- |
| **Agents of change and publicity campaigns** | Campaign using mass media, community activities and/or travel co-ordinator to promote alternative modes of transport | Unclear effect | 2+ |  |  |  |  |  |  |
| **Financial incentives** | Charging road users, e.g. road tolls, charging for car park space at work | Unclear effect. May depend on specifics of intervention | 2+ |  |  |  |  |  |  |
| **Provision of alternative services** | Neighbourhood-based car-sharing cooperative. | Unclear effect. Possibility of small increase in car use; will depend on specifics of intervention | 2- |  |  |  |  |  |  |
