# Supplementary material for: Assessing the unintended health impacts of road transport policies and interventions: translating research evidence for use in policy and practice
Source: BMC Public Health. 2008 Sep 30;8:339. doi: 10.1186/1471-2458-8-339 (PMC2567981; doi:10.1186/1471-2458-8-339)
Supplement: Additional file 4 — Table S4. Summary of the health and related impacts of new roads with indication of strength of research evidence (SoE). [file 1471-2458-8-339-S4.doc]

**Table 4: Summary of the health and related impacts of new roads with indication of strength of research evidence (SoE) ^[15]^**

|  | **Injuries** | **SoE** | **Respiratory health** | **SoE** | **Disturbance in affected area: noise, vibrations, fumes and dirt** | **SoE** | **Community severance** | **SoE** | **Evidence of impact displacement** | **SoE** |
| --- | --- | --- | --- | --- | --- | --- | --- | --- | --- | --- |
| **New major urban roads or road widening** | Little or no decrease in overall injury crashes across wider road network  Reduction in fatal crashes following addition of central overtaking lane to 2 lane road | 2++  2- |  |  | Increased disturbance due to noise | 2- | Neighbourhood traversal may fall immediately after new road building but residents adapt neighbourhood boundaries over time (1 study, 30 years effect still observed) | 3 | Displaced noise disturbance from relieved roads now with less traffic- relieved roads now quieter | 3 |
| **Bypasses** | Overall decrease in both old and new roads  Possibility of increased crashes where old and new road intersect | 2++  3 | Little or no improvement after one year. Possible small improvement for minor respiratory symptoms. | 2- | Increased noise and related sleep disturbance for those living near by-pass- little evidence of adaptation- greatest benefit for small towns. | 2++ | Decreased in area being bypassed. | 2++ | Possibility of displacement of injuries to secondary roads.  Disturbance factors displaced to area around by-pass | 4  2- |
| **Major connecting roads** | Overall decrease in injury crashes  Little evidence of change in crash severity | 2++  2++ |  |  |  |  |  |  |  |  |
