# Supplementary material for: Assessing the unintended health impacts of road transport policies and interventions: translating research evidence for use in policy and practice
Source: BMC Public Health. 2008 Sep 30;8:339. doi: 10.1186/1471-2458-8-339 (PMC2567981; doi:10.1186/1471-2458-8-339)
Supplement: Additional file 5 — Figure S1. Some possible pathways to health and related impacts following modal shift from driving to cycling to work. [file 1471-2458-8-339-S5.doc]

**Figure I: Some possible pathways to health and related impacts following modal shift from driving to cycling to work**

**Impact type: Physical activity Injury Environment & air quality**

**Impact level: *individual***

Increased physical activity Increased exposure to Lower roadside pollution may be lower

*(dependent on changes from baseline)* motorised traffic and risk of compared to in-vehicle concentrations

accident *(influenced by local* BUT increased inhalation caused by

*context, levels of cycling, and* physical exertion may increase exposure

*cyclist behaviour)* to harmful transport fuel pollution

Reduced obesity & cardio-

vascular risk *(dependent on* Uncertain health impacts *(for healthy*

*changes from baseline)* *individuals able to cycle to work)*

**Impact level: *local population & beyond***

Increased proportion of Improved air quality locally

cyclists using roads may *(dependent on significant*

reduce risk of injury for *reduction in transport related*

cyclists & pedestrians *fuel use)*

locally

Small improvement to cardio-

respiratory health locally and

beyond. Reduced cardiac &

respiratory related mortality,

especially among vulnerable groups.

**Impact level: *worldwide***

Reduced fuel emissions *(dependent*

*on significant reduction in transport*

*related fuel use)*

Reduced impact on climate

change at global level

Possible reduction in adverse health impacts

Modal shift from driving to cycling to work (suitability of cycling to work will be affected by distance to work, facilities to change into work clothes in the workplace, weather, other business (personal or work) to be carried out on journey to/from work e.g. shopping, taking children to school etc.
